# Perspective: Towards Personalised Metabolic Coaching in Cancer

**Published:** 2018-09

**Authors:** T Van Soom *, W Tjalma *, S El Bakkali, H Verbelen, N Gebruers, E van Breda

**Affiliations:** University of Antwerp, Faculty of Medicine and Health Sciences; Department of Rehabilitation Sciences and Physiotherapy, Research group MOVANT, Antwerp Multidisciplinary Research Unit (AM2RUN); Universiteitsplein 1 2610 Wilrijk. Belgium; Antwerp University Hospital (UZA), Multidisciplinary Edema Clinic; Wilrijkstraat 10 2650 Edegem. Belgium; Antwerp University Hospital (UZA), Multidisciplinary Breast Clinic, Wilrijkstraat 10 2650 Edegem. Belgium.

**Keywords:** Oncology, Energy Expenditure, Indirect Calorimetry, Metabolic Coaching

## Abstract

Although cancer survivorship has improved over the last decades, numbers of cancer incidence and prevalence are rising. Evidence is growing that lifestyle factors, such as physical activity, a healthy weight management and -diet, play an important role in first- and second line preventive strategies. When implementing a healthy lifestyle, the maintenance of the energy balance should be taken into account. The energy equilibrium is achieved when the energy intake (Ei) for one day is equal to the total daily energy expenditure (TEE). The latter is, among others, made up of the resting energy expenditure, its largest contributor (60-80% of TEE), and can be assessed by indirect calorimetry (i.e. the gold standard). The resting energy expenditure reflects the individual’s minimal caloric need in 24h to support basal functions. In cancer patients, energy imbalances, expressed as a positive (Ei > TEE) or negative (Ei & TEE) energy balance, may occur and are characterised by weight gain or -loss respectively. As a corollary, shifts in fatmass and fatfree mass are reported. Adequate nutritional follow-up is necessary in order to meet the energy needs, since both positive and negative energy balances are known to have deteriorating effects on cancer prognosis and mortality. In the clinical setting, predictive formulas (e.g. Harris-Benedict equation) are often used to estimate the caloric need. However, both under- and overfeeding are reported when using equations. Therefore, we advise to use indirect calorimetry in the standard assessment of a patient’s energy need in order to provide adequate metabolic coaching and -follow up.

## Importance of a healthy lifestyle in terms of body composition

Cancer survivorship has improved significantly as a result of screening, early detection and the development of novel targeted (personalised) therapies (Torre et al., 2016). However, the incidence and prevalence numbers of cancer are still growing (Ferlay et al., 2014). As estimated by the World Health Organisation, cancer has surpassed cardiovascular disease as the leading cause of death in developed countries (Ferlay et al., 2014; Vineis et al., 2014). Continuous Westernization through economic growth and social reforms in less developed countries, increase the likelihood of a growing cancer burden worldwide, causing a public health- and medical care problem (Ferlay et al., 2014; Mayne et al., 2016).

Only a small number of cancers are due to inherited conditions. Most cancers result from environmental issues and daily personal habits and manners of living. Besides primary prevention, cancer survivors show poorer health compared to the healthy population, thereby increasing the risk of cancer recurrence, and thus stressing the importance of second line prevention as well (Tan et al., 2019).

There is ample evidence that lifestyle factors, such as physical activity, a healthy weight management and -diet, play an important role in preventive strategies in cancer (Rock et al., 2012). Therefore, the implementation of a healthy lifestyle programme may reduce treatment-related side-effects and ameliorate overall functioning in activities of daily living (ADL) (Denlinger et al., 2016). Despite these beneficial effects on health, population-based studies showed that more than half of the cancer survivors do not meet the recommended guidelines for physical activity, and in the case of breast cancer (BC), about one third the survivors are obese or overweight (Tan et al., 2019).

## Body composition

Obesity and excess body fat are well-known risk factors for chronic diseases, and, in the case of cancer, a positive association with postmenopausal BC has been demonstrated previously (Baumgartner et al., 1995; Sheng et al., 2018). Even short-term weight gain in premenopausal women may increase the risk of developing BC, although the results are ambiguous (Sheng et al., 2018). Furthermore, obesity in patients with BC is found to be related with cancer recurrence and a higher cancer-related mortality (Sheng et al., 2018; Lauby-Secretan et al., 2016). Numerous studies have reported that body weight gain in BC occurs during the first year post-diagnosis. According to the Women’s Healthy Eating and Living (WHEL) study, patients receiving chemotherapy were 65% more likely to experience an increase in body weight (Sheng et al., 2018). In terms of body composition, weight gain was associated with a decrease in fat free mass (FFM), especially lean body mass, and increase in fat mass (FM), indicative for sarcopenia and sarcopenic obesity (Irwin et al., 2005).

The loss of FFM is a result of an imbalance in protein homeostasis, being protein catabolism exceeding protein synthesis. Causes are multifactorial and are associated with different pathological conditions such as sarcopenia, malnutrition and cachexia (Tsai, 2012). In contrast to cachexia, malnutrition leads to the preferential loss of FM over the loss of FFM. The latter can basically be counteracted by nutritional and/or physically active intervention programmes (Gullett et al., 2011). Cancer induced cachexia (CIC), however, is a wasting syndrome, characterized by loss of FFM, with or without loss of FM (Tsai, 2012). Both cachexia and malnutrition result in loss of total body weight, but unlike the latter, it is suggested that CIC is hard to reverse by nutritional interventions because of an altered metabolic state (Gullett et al., 2011).

In the overall healthy population, body weight changes are caused by an increased or decreased food intake and/or a reduction in energy expenditure through lower levels of physical activity as the most influencing factors (Demark-Wahnefried et al., 2001; Purcell et al., 2016). Thus, the imbalance between energy intake (Ei) and energy expenditure is responsible for the changes in in body composition, expressed as a gain or loss of FM or FFM (Hall et al., 2011). In cancer, however, changes in body composition (sarcopenia, sarcopenic obesity and/or cachexia) are predicting factors of treatment induced toxicity and mortality (Tsai, 2012). All together, it is important to ensure an adequate follow-up of the patient’s energy balance and body composition.

## Energy balance and energy expenditure

The energy equilibrium in humans is maintained through a balanced control of both Ei and total energy expenditure (TEE) (Westerterp, 2018). The TEE over a day is made up of three components: (1) Resting energy expenditure (REE), (2) activity energy expenditure (AEE) and the thermic effect of food digestion, known as (3) diet induced energy expenditure (DEE) (Demark-Wahnefried et al., 2001). The REE is the largest contributor of the TEE (approximating 60-80%) whereas the DEE and AEE are the most variable accounting for approximately 10% and 15 – 30% of the TEE, respectively (Heydenreich et al., 2017). Both a positive or negative energy balance may occur. A positive energy balance is characterised by weight gain and defined by Ei > TEE. On the other hand, weight loss can be attributed to a negative energy balance and is expressed as Ei < TEE (Mehta et al., 2015). To describe a person’s energy metabolic state, the REE, defined by the amount of energy that is used in 24 h without losing FFM, can accurately be measured by the gold standard, i.e. indirect calorimetry (IC) (Demark-Wahnefried et al., 2001). The energy required for life originates from the oxidation of food substrates into energy and heat (Lam et al., 2016; Schoffelen, 2017). Indirect calorimeters assess the energy expenditure by measuring gas exchange, i.e. the oxygen uptake and carbon dioxide production. It is called “indirect” because the caloric combustion rate is calculated from a measurement of oxygen uptake, and not from the direct release of heat by indirect calorimetry. Measuring of O 2 uptake and CO 2 production reflects the rate of cellular metabolism of carbohydrates, fats and proteins to produce energy (Schoffelen, 2017).

Other methods for determining REE are formula based calculations, such as the predictive equation of Harris-Benedict (HBEq), resulting in the predicted REE (REEPred) (Purcell et al., 2016). According to the HBEq, the measured REE of the majority of the healthy population is within 10% of the REEPred, and is considered normometabolic (REE = 90-110% of REEPred) (Mehta et al., 2015). Interestingly, both hyper- (>110% of REEPred) and hypometabolism (<90% of REEPred), as well as normometabolism, have been found in cancer patients (Chen et al., 1994). Although the HBEq is easy to use and often applied in clinical practice, it is not recommended for standard clinical assessment and dietary advice, since both over- and underfeeding have been reported (Frankenfield, 2013; Pirat et al., 2013).

## Energy balance and resting energy expenditure in cancer

Accurately measuring REE reflects the minimal caloric requirement to sustain basal energy metabolism which is subject to individual variations such as age, height, weight, sex and some physiological aspects (Purcell et al., 2016; Mehta et al., 2015). The leading factor responsible for the large heterogeneity, as observed between individuals, is the amount of FFM which explains 60-80% of the variances in REE (Müller et al., 2009, Nicolini et al., 2013). Overall, it has been shown that a higher REE is triggered by a larger amount of FFM (Müller et al., 2009).

In cancer patients, however, changes in REE should be interpreted with caution because of patho-physiological factors (tumour type, -size and -stage). Considerable evidence supports an increase in REE of 8-9% during the tumour-bearing state, due to the high metabolic demand (Baumgartner et al., 1995; Nguyen et al., 2016; Friesen et al., 2015). Furthermore, evidence is accumulating that not only the tumour itself, but also treatment-related side-effects, such as the onset of inflammatory processes, may underlie the increase in REE (Rusu et al., 2018; Vyas et al., 2014). Such an elevated REE seems (based upon HBEq) to be present in approximately 50% of all cancer patients and is considered as a determinant for cancer induced malnutrition. In combination with a reduced Ei, an increased energy expenditure will stimulate weight loss due to loss of FFM and/or FM, as seen in cancer cachexia (Baumgartner et al., 1995; Juinot et al., 2018).

On the other end of the energy metabolic spectrum, hypometabolism, is present in about one third of the cancer patients but the underlying causes are only scarcely investigated (Juinot et al., 2018). To date, two mechanisms have been proposed regarding a positive energy balance in cancer. The main hypothesis for a decrease in REE is related to changes in body composition. As mentioned above, REE is mainly depending on FFM, and in cancer obesity, the HBEq tends to overestimate REE in comparison to the REE as assessed by IC (Frankenfield, 2013). Additionally, hypometabolism might act as a compensatory mechanism in order to restore the energy imbalance due to rapid weight loss induced by malnutrition or underfeeding (Coutinho et al., 2018).

## The evolution of energy expenditure during chemotherapy

**Figure 1:**
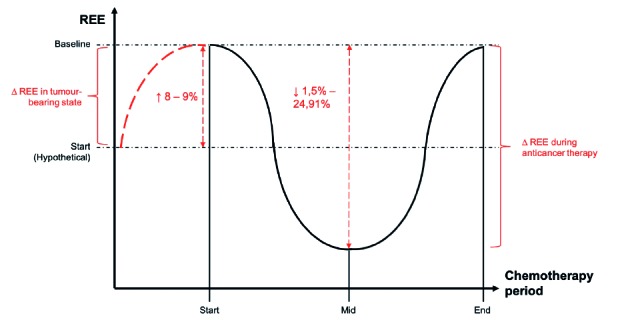
— Changes in resting energy expenditure REE (y-axis) = Resting energy expenditure; Chemotherapy period (x-axis) = Chemotherapy treatment period; ∆ REE = Change in REE; Start = Start treatment; Mid = Mid-treatment; End = End treatment; ↑ = Increase; ↓ = Decrease

The observed changes in REE during cancer treatment are still under debate but more evidence is accumulating that a U-shaped curve, with its nadir during mid-treatment and apex levels at the beginning and end of chemotherapy is found when REE is measured by IC (Figure 1). (Nguyen et al., 2016; Vyas et al., 2014). As mentioned above, literature has revealed that cancer patients during the tumour-bearing state experience an increase in REE of 8-9%. It can be hypothesised that a reduced tumour activity results in a decrease in energy expenditure. Such hypothesis is in line with previous results, where a significant decrease in REE has been reported in patients with complete remission of the tumour after chemotherapy treatment compared to non- or partial responders (Lerebours et al., 1988; Russell et al., 1984). Evidence that is indicative for the decrease in REE, comes from a study on surgical resections. In this study, a decrease in REE was noticed after curative removal of the tumour, and hence, the increasing effects of the tumour on energy expenditure were no longer present (Reeves et al., 2006). Another explanation for the initial decrease can be attributed to weight loss during the first part of the treatment (Juinot et al., 2018). A recent systematic review showed a decrease of approximately 1,5% - 25% in REE can be noticed, depending on tumour type and stage (Figure 1) (unpublished data, Van Soom, personal communication).

The second phase, the incremental section, of the U-shaped curve could be explained by the accumulative effects of chemotherapy due to inflammatory-induced complications (Garcia-Peris et al., 2005). Thus, there is evidence that cancer type, -stage, and type of chemotherapeutic drugs play an important role during the onset of inflammatory processes in cancer and cancer treatment (Figure 1). (Rusu et al., 2018; Vyas et al., 2014).

## The impact of a positive and negative energy balance on cancer

Energy metabolism varies over time. Both in the tumour-bearing state and treatment phase, fluctuations in REE have been noticed. Hypermetabolic patients, as well as hypometabolic patients represent a fragile group within the total cancer population, although both metabolic states are induced by different underlying mechanisms (Juinot et al., 2018).

On one end of the energy balance in the oncologic setting, cancer induced cachexia or malnutrition lurks around the corner. This catabolic state is, among other factors, characterized by hypermetabolism, resulting in a net negative energy balance and altered body composition (Tonorezos et al., 2013). The hypermetabolic response and the associated weight loss are related to a systemic inflammatory response with elevated levels of the inflammatory markers interleukin-6, tumour necrosis factor-alpha (TNF-alpha) and C-reactive protein. The latter, in turn, has been proven to be an independent predictor of early toxicity, affecting survival, prognosis and mortality (Juinot et al., 2018).

On the other end, a positive energy balance will manifest itself as overweight or obesity, as often seen in BC patients. The weight gain appears to reflect changes in body composition in which FFM (muscle mass) is exchanged by FM. Among cancer survivors, a positive energy balance, and thus weight gain, has a negative impact on relapses, recurrence and mortality (Demark-Wahnefried et al., 2001; Tonorezos et al., 2013).

All together both positive and negative energy balances are indicative for the need of a more detailed metabolic follow up, since this is highly likely to result in lower survival rates, lower outcomes in quality of life (QoL) and a higher mortality (Mehta et al., 2015; Douglas et al., 2007; Johnson et al., 2008).

## The use of indirect calorimetry

In a clinical setting the restoration of energy imbalances in cancer patients, are often based on equations like the HBEq. In the case of overweight or obese cancer patients, the HBEq tends to overestimate the REE whereas in malnourished patients the HBEq seems to underestimate the REE (Frankenfield, 2013; Pirat et al., 2009). To prevent over- and underfeeding, accurate analysis is of crucial importance in order to optimise nutritional support. Indirect calorimetry is generally accepted as the gold standard for measuring the caloric need. Unfortunately, it has not yet been widely applied in the clinical setting for economical and practical reasons (Pirat et al., 2009). Here we would like to stress the importance of adequate nutritional assessment by IC.

The main principle of IC is the collection of in- and expired gases under strict controlled conditions and adequate calibration by methanol combustion prior to a test and the use of span-gases and nitrogen every 15 minutes during operation (Schoffelen, 2017). A major step within IC is the conversion of the gases from the patient as Body Temperature Pressure Satured (BTPS) to the International accepted unit as Standard Temperature Pressure Dry (STPD) (Schoffelen, 2017). The biggest concern is the drying of the gases. We have taken special precautions but many, although user-friendly indirect calorimetric devices, lack accurate calibration and drying technology for physiological accurate indirect calorimetry measurement of energy expenditure.

## Personalised metabolic coaching in cancer - Take home message

Energy imbalances in general, and in cancer patients in particular, are related to a worse prognosis and higher mortality due to the underlying patho-physiological conditions. Besides, changes in REE can also be the result of the anticancer treatment itself. Although supported by a recent meta-analysis, it is important to keep in mind that the initial increase in REE must be interpreted with caution (Figure 1) (T. Van Soom, personal communication). In cancer, baseline measurements of REE are not true baseline measurements because of the presence of the tumour. This leaves us with a hypothetical REE at baseline. Since the true baseline values are not available in cancer patients, comparison with a patient’s physiological REE is nearly impossible. In order to obtain adequate information on energy expenditure for monitoring and providing accurate nutritional advice to the cancer patient, we strongly suggest the use of IC above the clinical often used HBEq to assess REE. In order to make correct statements on a cancer patients’ energy expenditure we also recommend to measure FFM (muscle mass) as it is the largest contributor to energy expenditure.

In conclusion, due to the continuing changes in disease state and therapeutic interventions which affect energy metabolism, an accurate assessment of REE by HBEq is questionable (Haugen et al., 2007). Therefore, measuring REE by IC will lead to a far more accurate evaluation of energy expenditure and the caloric needs in cancer patients. Besides, the clinical availability of IC has improved as a consequence of the development of less expensive equipment, such as portable devices (Haugen et al., 2007). A correct assessment of REE will lead to more individualised nutritional advice which, in turn, will support treatment and reduce long-term side effects.

## Take home messages

1. Lifestyle factors can only be successfully introduced to patients when personalised.
2. Personalised lifestyle factors will not only be beneficial in primary prevention but also in secondary preventive measures in cancer patients.
3. A large heterogeneity in the expression of REE is found. Therefore, indirect calorimetry is preferred over equations, like HBEq, to measure energy expenditure.
